# Complications and Disease Recurrence After Ileocecal Resection in Pediatric Crohn's Disease: A Retrospective Study

**DOI:** 10.1055/a-2048-7407

**Published:** 2023-04-11

**Authors:** M. Glenisson, A. Bonnard, D. Berrebi, N. Belarbi, J. Viala, C. Martinez-Vinson

**Affiliations:** 1 Department of Pediatric Surgery, Hôpital Universitaire Robert Debré, Assistance Publique Hôpitaux de Paris, Université de Paris, Paris, France; 2 Department of Pediatric Pathology, Hôpital Universitaire Robert Debré, Assistance Publique Hôpitaux de Paris, Université de Paris, Paris, France; 3 Department of Pediatric Radiology, Hôpital Universitaire Robert Debré, Assistance Publique Hôpitaux de Paris, Université de Paris, Paris, France; 4 Department of Pediatric Gastroenterology, Hôpital Universitaire Robert Debré, Assistance Publique Hôpitaux de Paris, Université de Paris, Paris, France

**Keywords:** Crohn's disease, ileocecal resection, postoperative recurrence

## Abstract

**Objective** The aim of this retrospective study was to describe the risk of postoperative recurrence (POR) after ileocecal resection, the occurrence of surgical complications, and identify predictors of these adverse postoperative outcomes in pediatric Crohn's disease (CD).

**Patients and methods** All the children less than 18 years of age with a diagnosis of CD, who underwent primary ileocecal resection for CD between January 2006 and December 2016 in our tertiary center, were considered for inclusion. Factors related to POR were investigated.

**Results** A total of 377 children were followed for CD between 2006 and 2016. During this period, 45 (12%) children needed an ileocecal resection. POR was diagnosed in 16% ( *n* = 7) at 1 year and 35% ( *n* = 15) at the end of the follow-up, with a median follow-up of 2.3 years (Q1–Q3 1.8–3.3). Median duration of the postoperative clinical remission was 1.5 years (range 0.5–2). Multivariate Cox regression analysis identified only young age at diagnosis as a risk factor for POR.

In total, 7 of the 43 patients (16%) developed severe postoperative complications, defined as requiring surgical, endoscopic, or radiological intervention. The only risk factor was intraoperative abscess.

**Conclusion** Only young age at diagnosis was associated with POR. This information could be useful to develop targeted therapeutic strategies for young CD children. At the end of follow-up with a median follow-up of 2.3 years (Q1–Q3 1.8–3.3), there was no surgical POR: endoscopic dilatation for POR should be considered in order to delay or prevent surgery.

## Introduction

The clinical course of childhood-onset Crohn's disease (CD) is more severe with more extensive disease, more aggressive disease behavior, and an earlier exposure to major postsurgical sequelae. 1 By 5 years, 14 to 47% of pediatric CD patients require surgery, and the most common surgery is ileocecal resection with a range of 44 to 71%. 2 Surgery may be considered as an alternative to medical therapy when a patient has active disease limited to a short segment despite optimized medical treatment, and also in children in the prepubertal or pubertal stage if growth velocity for bone age is reduced over a period of 6 to 12 months despite optimized medical and nutritional therapy. 3

Surgical resection is not curative for CD. Relapse, both at the area of anastomosis and at other sites, frequently occurs within 5 years postsurgery. 4 5 6

The postoperative recurrence (POR) rate in adults varies according to the definition used, be it clinical, endoscopic, radiological, or surgical. Smoking, prior intestinal surgery, absence of prophylactic treatment, penetrating disease at index surgery, perianal location, granulomas in the resection specimen, and myenteric plexitis are considered predictors of early POR after ileocolic resection. 7

In pediatric CD, clinical POR varies from 18 8 to 50% 6 at 1 year and from 34% 9 to 73% 6 at 5 years. Diffuse ileocolic disease, penetrating and stricturing disease, severe disease, complex disease, age less than 14 years at diagnosis, and failure of medical management increase the risk for recurrence. 2

The aim of this study was to describe the risk of POR after ileocecal resection, the occurrence of surgical complications, and identify predictors of these adverse postoperative outcomes in pediatric CD.

## Methods

### Patients

All children under 18 years of age with a diagnosis of CD according to the revised Porto criteria—based on a combination of history, physical and laboratory examination, esophagogastroduodenoscopy and ileocolonoscopy with histology, and imaging of the small bowel 10 —who underwent primary ileocecal resection for CD between January 2006 and December 2016 in our tertiary center, were considered for inclusion in this retrospective study.

Patients were collected using the hospital medicoadministrative database *Programme de Médicalisation des Systèmes d'Information* and the national register CEMARA ( *CEntre des MAladies RAres* ) for our center. 11

The extraction with these two information systems allowed the selection of all the children under 18 years of age with a diagnosis of CD, who were followed between January 2006 and December 2016 in our tertiary center.

The study was approved by the Institutional Review Boards for ethical approval and the French national data protection agency (Commission Nationale de l'informatique et des libertés n°19811722). All patients were informed.

### Data Collection

Data were retrospectively obtained from medical files and recorded in an anonymous standardized form. The following baseline characteristics were recorded for each patient: gender, age at diagnosis, CD classification according to Paris classification 12 and disease activities according to Pediatric Crohn's Disease Activity Index (PCDAI) score. 13 The Paris classification, emerged in 2011, in order to address gaps attributed to the dynamic features of pediatric disease phenotype, including the change in disease location and behavior over time, and the effect of the disease on growth over time, classifying age at diagnosis as A1a (0 to < 10 years), A1b (10 to < 17 years), A2 (17 to 40 years), and A3 (> 40 years), distinguishing disease above the distal ileum as L4a (proximal to ligament of Treitz) and L4b (ligament of Treitz to above distal ileum), allowing both stenosing and penetrating disease to be classified in the same patient (B2B3), and denoting the presence of growth failure in the patient at any time as G _1_ versus G _0_ (never growth failure). The PCDAI score incorporates clinical characteristics (stool frequency, abdominal pain, well-being, extraintestinal manifestations, abdominal mass, perirectal disease, body weight, and height velocity score) and routine laboratory tests (erythrocyte sedimentation rate, hematocrit, and albumin). It ranges from 0 to 100, with the highest scores indicating an active disease: a PCDAI of 10 or lower defined clinical remission while scores between 11 and 30 and over 30 defined, respectively, low to moderate or severe activity.

Pre- and postoperative data included age at surgery, disease activity (PCDAI), the use of CD-related medication, endoscopy, and radiographic imaging such as ultrasound or magnetic resonance imaging (MRI). Preoperative data were collected at the preoperative workup. Postoperative data were collected at the last visit without recurrence and at the recurrence. Pubertal status was not available. Surgical variables included the type of surgical approach, type of anastomosis, additional surgical procedures, stoma placement, operating time, pathology report of the resected segment, and postoperative complications according to Dindo et al. 14

## Outcomes

### Complications

Severe postoperative complications were defined as requiring surgical, endoscopic, or radiological intervention (Dindo et al). 14 Intra-abdominal septic complications (IASCs) were defined as anastomotic leak, enterocutaneous fistula, and intra-abdominal abscesses de novo. Risk factors for IASC were investigated.

### Disease Postoperative Recurrence

In our study, the POR of CD was defined as an increase in the PCDAI of 12.5 points 15 accompanied by either radiological (ultrasound or MRI) or endoscopic confirmation of recurrent CD. PCDAI was collected postoperatively at each visit (every 3 months). Radiological recurrence was defined as increased gut wall thickness and increased vascular pattern or echogenicity (ultrasound) or contrast enhancement (MRI) extending from the anastomosis proximally, either in ultrasound or in MRI. 16 17 Surgical POR was defined as recurrence requiring a new surgery. Risk factors for POR were explored. Endoscopic recurrence was described using the Rutgeerts score. Rutgeerts score is used as the standard evaluation of postsurgical recurrences at the ileocolic anastomosis level. The risk of having symptoms of the disease within 5 years after a postsurgical control endoscopy changes from 6% of the patients classified as i0 and i1 to 27% of patients classified as i2, 63% for patients i3, and 100% for subjects i4. I1 is defined as not more than five anastomotic aphthous lesions in the distal ileum, i2 as more than five aphthous lesions with normal mucosa between the lesions, or skip areas of larger lesions or ulcers up to 1 cm confined to ileocolonic anastomosis, and i3 as diffuse aphthous ileitis with diffusely inflamed mucosa between the multiple aphthae and i4 as diffuse inflammation, with larger lesions: large ulcers and/or nodules/cobble and/or narrowing/stenosis. 18

### Statistical Analysis

Categorical variables were described as frequencies and percents. Continuous variables were described as the median (interquartile range), means, and standard deviations).

Univariate analyses were performed using the nonparametric Mann–Whitney test for continuous variables and the chi-square test or Fischer's exact test as appropriate for categorical variables. Predictors for POR were identified by multivariate Cox regression analysis.

Eight variables were chosen a priori based on the literature review: age at diagnosis, L4 location, penetrating behavior, perianal disease, granulomas in resection specimen and myenteric plexitis and inflammation of the ileocecal valve, and treatment with biologics after the first surgery. 7 19

A *p* level less than 0.05 was considered significant.

Analyses were conducted using RStudio 1 (Rstudio, Inc).

## Results

### Ileocecal Resection

**Fig. 1:**
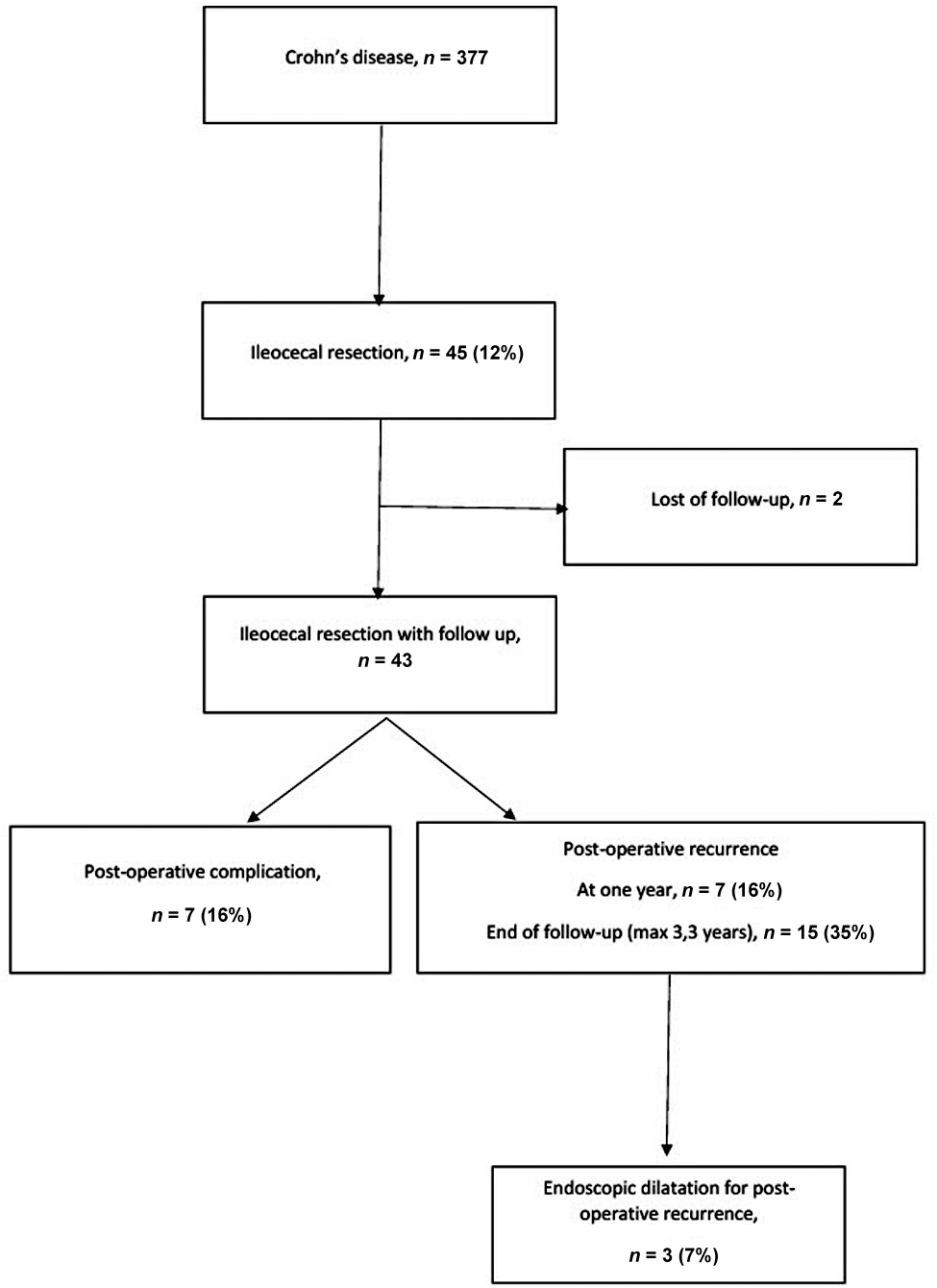
Flow chart.

A total of 377 children were followed for CD at Robert Debré hospital between 2006 and 2016 ( Fig. 1 ). During this period, 45 (12%) children needed an ileocecal resection for CD. The laparoscopic approach was used in all cases for mobilization of the ileocaecal region. In the total laparoscopic approach ( *n* = 16, 37%), an intracorporeal ileocolic anastomosis was performed, and in the laparoscopic-assisted approach ( *n* = 27, 63%), an extracorporeal anastomosis was performed. In the latter, access was obtained by either a McBurney, midline, or Pfannenstiel incision

Follow-up was available for 43, with a median follow-up of 2.3 years (Q1–Q3 1.8–3.3). Sixteen (37%) ileocecal resections were done before 2011 and twenty-seven (63%) after. The indications for ileocecal resection were biologics therapy refractory inflammation (49%, *n* = 22), intra-abdominal abscesses (24.4%, *n* = 11), active disease limited to a short segment associated with growth delay (13.3%, *n* = 6), surgery rather than anti-TNF (11.1%, *n* = 5; patient's refusal of anti-TNF), and digestive perforation (2.2%, *n* = 1). Biologics therapy refractory inflammation was defined as a lack of response leading to surgery. In this group of 22 patients, all received anti-TNF, 11 patients received more than one biological agent (nine changed twice and two changed three times). The median duration of biological treatment was 18 months (Q1–Q3: 7–21.5). Table 1 depicts patients' demographic and surgical characteristics at the time of ileocecal resection and details regarding the surgery.

**Table 1:** Demographic and surgical characteristics of patients at time of ileocecal resection

Characteristics	Total *n* = 43	Recurrence *n* = 15	No recurrence *n* = 28	*p-* Value
Female, *n* (%)	23 (51)	7 (47)	16 (57)	0.5
Age at surgery mean (± SD)	15.19 (1.76)	14.47 (1.99)	15.57 (1.53)	0.05
Years since diagnosis median (Q1–Q3)	2 (0.5–4)	3.3 (1.5–4.5)	1.8 (0.4–3.3)	0.15
Age at diagnosis median (Q1–Q3)	12 (10.5–15)	11 (9.5–14)	14 (11–16)	0.05
Paris classification, *n* (%)				
A1a	7 (16)	4 (27)	3 (11)	
A1b	36 (84)	11 (73)	25 (89)	
Disease location, *n* (%) at diagnosis				
L1	30 (69)	8 (53)	22 (79)	0.16
L2	0 (0)	0 (0)	0 (0)	
L3	13 (31)	7 (47)	6 (21)	
L4	13 (33)	7 (54)	6 (23)	0.07
Disease behavior, *n* (%)				
B1	2 (5)	2 (13)	0 (0)	0.17
B2	26 (60)	9 (60)	17 (61)	
B3 or B2B3	15 (36)	4 (27)	11 (39)	0.50
Perianal location, *n* (%)	2 (5)	2 (14)	0 (0)	0.11
Extraintestinal manifestations, *n* (%)	7 (18)	3 (21)	4 (15)	
Intestinal occlusion, *n* (%)	4 (9)	1 (7)	3 (11)	
Medical therapy before surgery, *n* (%)				
Steroids	12 (28)	3 (20)	9 (32)	0.5
Immunosuppressive therapy	19 (44)	6 (40)	13 (46)	0.4
Biologics	24 (56)	9 (60)	15 (54)	0.3
Antibiotics	18 (42)	8 (53)	10 (36)	0.5
Medical therapy after surgery, *n* (%)	42 (98)	15 (100)	27 (96)	
Immunosuppressive therapy	25 (58)	8 (53)	17 (61)	
Biologics	17 (40)	7 (47)	10 (36)	0.5
Laboratory				
C-reactive protein > 10 mg/L, *n* (%)	31 (76)	13 (93)	18 (67)	0.12
Albuminemia < 30 g/L, *n* (%)	15 (36)	8 (53)	7 (26)	0.10
Hemoglobin < 11 g/dL	16 (38)	6 (43)	10 (36)	
Surgical access, *n* (%)				
Laparoscopy	16 (37)	5 (33)	11 (39)	
Assisted laparoscopy	27 (63)	10 (66)	17 (61)	
Type of anastomosis, *n* (%)				
Side to side	30 (70)	10 (67)	20 (71)	
Stapled	19 (44)	5 (33)	14 (50)	0.4
Intracorporeal	18 (42)	5 (33)	13 (46)	0.5
Protective stoma, *n* (%)	3 (7)	1 (7)	2 (7)	
Operating time, median (Q1–Q3), min	175 (127–214)	174 (123–212)	175 (129–212)	
Length of ileal resection > 30 cm				
*n* (%)	7 (17)	4 (27)	3 (11)	0.2
Length of stay after surgery				
Median (Q1–Q3), days	7 (6–12)	9 (6–12.5)	7 (6–12)	0.7
Postoperative complications, *n* (%)	7 (16)	3 (20)		

The 11 patients with an abscess were treated with antibiotics before surgery, 4 only with enteral nutrition and antibiotics, 2 with immunosuppressants and antibiotics, 3 with enteral nutrition, immunosuppressant, and antibiotics, and 2 with anti-TNF and antibiotics. Nine patients underwent ileocecal resection without complication and surgery resolved the abscess. An intraoperative abscess complicated the ileocecal resection for the two patients treated with anti-TNF before surgery, requiring a protective per operative stoma placement.

Surgery was performed for growth delay associated with active disease for six patients. Three patients were receiving enteral nutrition before surgery. Height velocity (cm/y) increased from 1.1 cm/y (Q1–Q3: 0–3.3) to 5.8 cm/y (Q1–Q3: 3.1–8.4) after surgery.

One patient had a digestive perforation with sepsis requiring a stoma 9 months before the ileocecal resection.

During surgery three patients required a protective stoma placement: two patients for an intraoperative abscess and one patient for a severe digestive and joint disease requiring heavy immunosuppressive drugs (steroids and anti-TNF and methotrexate).

Patients received systematically perioperative antibiotics. Immunosuppressants were delayed after surgery with a median of 12 days (Q1–Q3: 6–34).

### Severe Postoperative Complications

Seven of the forty-three patients (16%) developed severe postoperative complications ( Fig. 1 ). One patient developed anastomotic leak requiring a stoma, and six intra-abdominal abscess de novo after resection including the two intraoperative abscess. The only risk factor for IASC was intraoperative abscess. Risk factors for IASC are analyzed in Table 2 .

**Table 2:** Univariate analysis: risk factors for intra-abdominal septic complications

Characteristics	No postoperative complication *n* = 36	Postoperative complication *n* = 7	*p* -Value
Age at surgery, mean (± SD), years	15.06 (1.84)	15.86 (1.21)	0.28
Preoperative biologics, n (%)	18 (50)	6 (86)	0.11
Preoperative steroids, *n* (%)	11 (31)	1 (14)	0.6
Disease behavior B3 or B2B3, *n* (%)	8 (22)	3 (43)	0.35
Albumin < 30 g/L, *n* (%)	12 (33)	3 (43)	0.7
CRP > 10 mg/L, *n* (%)	26 (72)	5 (71)	
Intracorporeal anastomosis, *n* (%)	13 (36)	5 (71)	0.11
Intraoperative abscess, *n* (%)	0 (0)	2 (5.5)	0.02
Operating time, median (Q1–Q3), min	174 (124–213)	197 (146–218)	0.5

Abbreviation: CRP, C-reactive protein.

### Disease Postoperative Recurrence

POR was diagnosed in 16.3% ( *n* = 7) at 1 year and 35% (n = 15) at the end of the follow-up, median 2.3 years (range 1.8–3.3) ( Table 1 and Fig. 1 ). Median duration of the postoperative clinical remission was 1.5 years (range 0.5–2). The median increase in PCDAI was 20 (Q1–Q3: 15–35). An endoscopy was available for nine patients. Proximal spread of disease was seen in four patients (i3 *n* = 4). A flare-up on the colonic side was also seen in two of these four patients. In five patients, lesions were confined to the ileocolonic anastomosis with stenosis in three (i2 *n* = 2, i4 *n* = 3). For six patients, clinical recurrence was confirmed by radiology, showing an increased gut wall thickness extending from the anastomosis proximally. At the time of ileocecal resection, 44% ( *n* = 19) were treated with immunosuppressive therapy, 40% ( *n* = 6) in the POR group and 46% ( *n* = 13) in the no POR group. In total, 56% ( *n* = 24) were treated with biologics, 60% ( *n* = 9) in the POR group, and 54% ( *n* = 15) in the no POR group.

After resection, only one patient was just followed up, 25 patients (58%) were treated with immunosuppressive therapy (azathioprine *n* = 18, 6 mercaptopurine *n* = 4, and methotrexate *n* = 3), and 17 (40%) with biologics (anti-TNF *n* = 15, ustekinumab *n* = 2).

### Fecal Calprotectin was not Available during Follow-up

Endoscopy was performed only for 19 (9 symptomatic and 10 asymptomatic) patients at a median of 14 months after surgery (Q1–Q3: 5–20.5), at the time of clinical recurrence for 9 patients. Only two patients had endoscopic remission and 89% endoscopic recurrence ( *n* = 17, endoscopic AND clinical recurrence *n* = 9, only endoscopic recurrence with no symptoms *n* = 8; Rutgeerts score > i2).

At the end of follow-up, there was no surgical POR. Three patients (7%) needed an endoscopic balloon dilatation for POR during follow-up (min 25 months and max 82 months) for anastomotic stricture. The first patient developed an anastomotic stricture 2 years after the ileocecal resection. He underwent three endoscopic balloon dilatations (every 2 months), and medical treatment was also changed (azathioprine replaced by anti-TNF, replaced by ustekinumab). At the last endoscopic control, 5 years after the ileocecal resection, no stricture was found. The second patient developed an anastomotic stricture 3 years after the ileocecal resection, but he was initially asymptomatic. Seven years after the ileocecal resection, endoscopic balloon dilatation was performed for Koenig's syndrome, and treatment was changed (ustekinumab replaced by anti-TNF). As patient became asymptomatic, no more dilatation was performed with a follow-up of 3 years. The last patient developed an anastomotic stricture 3 years after the ileocecal resection. Three endoscopic balloon dilatations were performed every 6 months with no results. Surgery is actually under discussion (8 years after the ileocecal resection).

Multivariate Cox regression analysis identified only young age at diagnosis as a risk factor for POR ( Table 3 ). The median age at diagnosis was 11 years for patients with POR and 14 years for patients without POR ( *p* = 0.05). Surgery was performed within a median of 3.3 years after CD diagnosis for POR patients and 1.8 years for those without POR.

**Table 3:** Multivariate Cox regression analysis: risk factors for postoperative recurrence

	HR	95% CI	*p* -Value
Age at diagnosis	0.73	0.54–0.98	0.04
Disease location L4	4.11	0.70–24.04	0.12
Disease behavior B3 or B2B3	3.06	0.42–22.34	0.27
Perianal location	1.33	0.52–3.28	0.55
granulomas in resection specimen	0.73	0.11–4.71	0.74
Myenteric plexitis in resection specimen	3.21	0.55–18.66	0.19
Inflammation of the ileocecal valve in the resection specimen	0.75	0.22–2.53	0.64
Postoperative biologics	0.44	0.08–2.30	0.20

Abbreviations: CI, confidence interval; HR, hazard ratio.

## Discussion

In this retrospective study, 12% of CD patients needed ileocecal resection. To our knowledge, only three studies report ileocecal resection in children alone and not intestinal resections of different extents and locations 20 21 22 but focused on POR rates. The rate of primary surgery in literature is variable: from a cumulative incidence of 5 to 7% at 1 year, 15 to 20% at 5 years, and 30 to 34% at 5 years 23 24 25 in pediatric CD. The rate of primary surgery in our center was in the same range.

The cohort considered was uniformly diagnosed and uniformly treated. ESPGHAN recommendations stating that “anti-TNF therapy as primary induction therapy may be considered for selected children at high risk for poor outcome” were adopted in our center after 2018. 26 Only 24 patients (56%) were treated with anti-TNF at the time of ileocecal resection.

POR was observed in 16.3% of patients at 1 year and 35% at the end of follow-up (median 2.3 years [range 1.8–3.3]), with a median duration of remission for 1.5 years (range: 0.5–2). In other pediatric series, ileocecal resection resulted in clinical recurrence of 20% of the patients at 1 year and 44% during the entire follow-up of 30 months. 20 For Diederen et al, clinical recurrence was observed in 49.2% of subjects at 5 years from resection. 22 Clinical disease recurrence was previously reported in 65% of adult patients 25 and in 60% of pediatric patients 27 at 5 years from ileocecal resection. Our recurrence rate was slightly lower from other series, but we defined POR as an increase in the PCDAI of 12.5 points 15 accompanied by either radiological or endoscopic confirmation of recurrent CD. The objective of the radiologic confirmation was to confirm recurrence. Functional abdominal disorders can indeed increase PCDAI (abdominal pain and diarrhea). Fecal calprotectin was available only for a few patients. To circumvent this methodological difficulty, we add radiological data to our POR definition. In this cohort, 44% of patients received immunosuppressive therapy and 56% biologics before surgery, and 58 and 40% after. Patients receiving immediate postoperative therapy are known to develop less clinical and surgical recurrence. A recent systematic review and meta-analysis reported that after the year 2000, the surgical rates at 1, 5, and 10 years have significantly decreased. It is unclear whether this decrease can be solely attributable to the introduction of biologics, the more effective use of thiopurine dosing with monitoring or perhaps earlier disease recognition and early use of effective interventions. 28

Despite the small sample size, endoscopic recurrence rate was 38% at 6th month for Zarubova et al. 21 Bobanga et al reported a recurrence rate of 87% in patients younger than 16 years who underwent endoscopy during mean 3 years follow-up compared to 70% in patients older than 16. 29 In our series, endoscopy was performed only for 19 patients during long-term follow-up. Only two patients had endoscopic remission and 89% endoscopic recurrence. Moreover, endoscopy was in most cases performed after clinical relapse (68%). It is an important limitation of our study: endoscopy should have been performed systematically as recommended. Indeed, endoscopy is the standard for evaluation of POR, and endoscopic assessment within 1 year of surgical resection is recommended. Endoscopic recurrence precedes the onset of clinical symptoms. It is important to identify asymptomatic patients at risk for clinical recurrence and disease complications with early endoscopic surveillance. 2

In this retrospective study, the inconsistent use of endoscopy was a weakness. Nevertheless, MRI is better accepted than endoscopy by children, is less invasive, and has shown reliable accuracy to detect endoscopic activity in CD patients. 17 30 We use MRI for the evaluation of the postoperative course of CD, dependent on our center's experience/availability, and patient preference.

At the end of follow-up, there was no surgical POR, three patients (7%) needed an endoscopic dilatation for POR, whereas surgical recurrence was observed in 11.9% of subjects in other pediatric series. 22 The aims of endoscopic treatment are to relieve the obstruction and obstruction-associated symptoms, delay or prevent surgery, 31 thereby preserving the bowel from chronic intestinal failure. 32

We found an association between young age at disease onset and at surgery and POR: the median age at diagnosis was 11 for POR patients and 14 for no POR patients ( *p* = 0.05). L4 location at diagnosis was present in 7 (54%) patients with POR and in 6 (23%) patients without, *p* = 0.07. Boualit et al found that young age (less than 14 years) at diagnosis, complicated behavior, and an L4 location were risk factors for undergoing second surgery; moreover, a first intestinal resection performed within 3 years after diagnosis was associated with reduced need for further IS and/or anti-TNF-a treatment and a better catch-up of height and weight. We found that surgery was performed within a median of 3.3 years after CD diagnosis for POR patients and 1.8 years for those without POR. Delaying surgery could also be associated with a poor outcome.

CD in children is known to be more severe and more aggressive. 1 Young age is a risk factor for disease severity. Young patients less than 14 and even more if less than 11 should be more monitored. Delaying surgery after 3 years could be a factor of poor outcomes. The occurrence of severe complications in this cohort was 16%, and all were infectious. This is more than previous reports in adults (11–13%) 33 34 and in children and adolescents (10–12%). 4 22 The only risk factor for IASC was an intraoperative abscess. In our cohort, in which 56% of patients received biologics before and 40% after the first surgery, biologics had no impact on IASC.

In conclusion, only young age at diagnosis was associated with POR. This information could be useful to develop targeted therapeutic strategies for young CD children less than 14 and more specifically less than 11. Surgery should not be delayed in order to prevent the poor outcome. At the end of follow-up, there was no surgical POR: endoscopic dilatation for POR should be considered to delay or prevent surgery.

## References

[1] Pigneur B Seksik P Viola S Natural history of Crohn's disease: comparison between childhood- and adult-onset disease Inflamm Bowel Dis 2010 16 06 953 961 19834970 10.1002/ibd.21152

[2] Splawski J B Pffefferkorn M D Schaefer M E NASPGHAN Clinical report on postoperative recurrence in pediatric Crohn disease J Pediatr Gastroenterol Nutr 2017 65 04 475 486 28937552 10.1097/MPG.0000000000001606

[3] IBD Working Group of ESPGHAN (IBD Porto Group) Amil-Dias J Kolacek S Turner D Surgical management of Crohn disease in children: guidelines from the Paediatric IBD Porto Group of ESPGHAN J Pediatr Gastroenterol Nutr 2017 64 05 818 835 28267075 10.1097/MPG.0000000000001562

[4] Blackburn S C Wiskin A E Barnes C Surgery for children with Crohn's disease: indications, complications and outcome Arch Dis Child 2014 99 05 420 426 24395646 10.1136/archdischild-2013-305214

[5] Piekkala M Pakarinen M Ashorn M Rintala R Kolho K L Long-term outcomes after surgery on pediatric patients with Crohn disease J Pediatr Gastroenterol Nutr 2013 56 03 271 276 23114471 10.1097/MPG.0b013e318279871c

[6] Hansen L F Jakobsen C Paerregaard A Qvist N Wewer V Surgery and postoperative recurrence in children with Crohn disease J Pediatr Gastroenterol Nutr 2015 60 03 347 351 25373863 10.1097/MPG.0000000000000616

[7] ECCO Gionchetti P Dignass A Danese S 3rd European evidence-based consensus on the diagnosis and management of Crohn's disease 2016: Part 2: surgical management and special situations J Crohn's Colitis 2017 11 02 135 149 27660342 10.1093/ecco-jcc/jjw169

[8] Pacilli M Eaton S Fell J M Rawat D Clarke S Haddad M J Surgery in children with Crohn disease refractory to medical therapy J Pediatr Gastroenterol Nutr 2011 52 03 286 290 20975579 10.1097/MPG.0b013e3181e999af

[9] Boualit M Salleron J Turck D Long-term outcome after first intestinal resection in pediatric-onset Crohnʼs disease Inflamm Bowel Dis 2013 19 7 14 22573565 10.1002/ibd.23004

[10] European Society of Pediatric Gastroenterology, Hepatology, and Nutrition Levine A Koletzko S Turner D ESPGHAN revised porto criteria for the diagnosis of inflammatory bowel disease in children and adolescents J Pediatr Gastroenterol Nutr 2014 58 06 795 806 24231644 10.1097/MPG.0000000000000239

[11] CEMARA task force Landais P Messiaen C Rath A CEMARA an information system for rare diseases Stud Health Technol Inform 2010 160(Pt 1):481 485 20841733

[12] Levine A Griffiths A Markowitz J Pediatric modification of the Montreal classification for inflammatory bowel disease: the Paris classification Inflamm Bowel Dis 2011 17 06 1314 1321 21560194 10.1002/ibd.21493

[13] Hyams J S Ferry G D Mandel F S Development and validation of a pediatric Crohn's disease activity index J Pediatr Gastroenterol Nutr 1991 12 04 439 447 1678008

[14] Dindo D Demartines N Clavien P A Classification of surgical complications: a new proposal with evaluation in a cohort of 6336 patients and results of a survey Ann Surg 2004 240 02 205 213 15273542 10.1097/01.sla.0000133083.54934.ae PMC1360123

[15] Griffiths A M Otley A R Hyams J A review of activity indices and end points for clinical trials in children with Crohn's disease Inflamm Bowel Dis 2005 11 02 185 196 15677913 10.1097/00054725-200502000-00013

[16] Alison M Kheniche A Azoulay R Roche S Sebag G Belarbi N Ultrasonography of Crohn disease in children Pediatr Radiol 2007 37 11 1071 1082 17899062 10.1007/s00247-007-0559-1

[17] Baillet P Cadiot G Goutte M Faecal calprotectin and magnetic resonance imaging in detecting Crohn's disease endoscopic postoperative recurrence World J Gastroenterol 2018 24 05 641 650 29434453 10.3748/wjg.v24.i5.641 PMC5799865

[18] Rutgeerts P Geboes K Vantrappen G Beyls J Kerremans R Hiele M Predictability of the postoperative course of Crohn's disease Gastroenterology 1990 99 04 956 963 2394349 10.1016/0016-5085(90)90613-6

[19] Buisson A Chevaux J-B Allen P B Bommelaer G Peyrin-Biroulet L Review article: the natural history of postoperative Crohn's disease recurrence Aliment Pharmacol Ther 2012 35 06 625 633 22313322 10.1111/j.1365-2036.2012.05002.x

[20] Hojsak I Kolacek S Hansen L F Long-term outcomes after elective ileocecal resection in children with active localized Crohn's disease—a multicenter European study J Pediatr Surg 2015 50 10 1630 1635 25913894 10.1016/j.jpedsurg.2015.03.054

[21] Zarubova K Hradsky O Copova I Endoscopic recurrence 6 months after ileocecal resection in children with Crohn disease treated with azathioprine J Pediatr Gastroenterol Nutr 2017 65 02 207 211 28248209 10.1097/MPG.0000000000001470

[22] Diederen K de Ridder L van Rheenen P Complications and disease recurrence after primary ileocecal resection in pediatric Crohnʼs disease Inflamm Bowel Dis 2017 23 272 282 28079626 10.1097/MIB.0000000000000999

[23] Gupta N Bostrom A G Kirschner B S Presentation and disease course in early- compared to later-onset pediatric Crohn's disease Am J Gastroenterol 2008 103 08 2092 2098 18796101 10.1111/j.1572-0241.2008.02000.x PMC3258513

[24] Schaefer M E Machan J T Kawatu D Factors that determine risk for surgery in pediatric patients with Crohn's disease Clin Gastroenterol Hepatol 2010 8 09 789 794 20566311 10.1016/j.cgh.2010.05.021

[25] Vernier-Massouille G Balde M Salleron J Natural history of pediatric Crohn's disease: a population-based cohort study Gastroenterology 2008 135 04 1106 1113 18692056 10.1053/j.gastro.2008.06.079

[26] European Crohn's and Colitis Organisation European Society of Pediatric Gastroenterology, Hepatology and Nutrition Ruemmele F M Veres G Kolho K L Consensus guidelines of ECCO/ESPGHAN on the medical management of pediatric Crohn's disease J Crohn's Colitis 2014 8 10 1179 1207 24909831 10.1016/j.crohns.2014.04.005

[27] Baldassano R N Han P D Jeshion W C Pediatric Crohn's disease: risk factors for postoperative recurrence Am J Gastroenterol 2001 96 07 2169 2176 11467649 10.1111/j.1572-0241.2001.03876.x

[28] Dubinsky M Have we changed the natural history of pediatric Crohn's disease with biologics? Dig Dis 2014 32 04 360 363 24969280 10.1159/000358137

[29] Bobanga I D Bai S Swanson M A Factors influencing disease recurrence after ileocolic resection in adult and pediatric onset Crohn's disease Am J Surg 2014 208 04 591 596 25110291 10.1016/j.amjsurg.2014.06.008

[30] Koilakou S Sailer J Peloschek P Endoscopy and MR enteroclysis: equivalent tools in predicting clinical recurrence in patients with Crohn's disease after ileocolic resection Inflamm Bowel Dis 2010 16 02 198 203 19504611 10.1002/ibd.21003

[31] Shen B Kochhar G Navaneethan U Practical guidelines on endoscopic treatment for Crohn's disease strictures: a consensus statement from the Global Interventional Inflammatory Bowel Disease Group Lancet Gastroenterol Hepatol 2020 5 04 393 405 31954438 10.1016/S2468-1253(19)30366-8

[32] Elriz K Palascak-Juif V Joly F Crohn's disease patients with chronic intestinal failure receiving long-term parenteral nutrition: a cross-national adult study Aliment Pharmacol Ther 2011 34 08 931 940 21848855 10.1111/j.1365-2036.2011.04806.x

[33] Alves A Panis Y Bouhnik Y Pocard M Vicaut E Valleur P Risk factors for intra-abdominal septic complications after a first ileocecal resection for Crohn's disease: a multivariate analysis in 161 consecutive patients Dis Colon Rectum 2007 50 03 331 336 17252288 10.1007/s10350-006-0782-0

[34] Brouquet A Bretagnol F Soprani A Valleur P Bouhnik Y Panis Y A laparoscopic approach to iterative ileocolonic resection for the recurrence of Crohn's disease Surg Endosc 2010 24 04 879 887 19730944 10.1007/s00464-009-0682-1

